# Highlands and Islands Medical Service Board

**Published:** 1918-07-20

**Authors:** 


### HIGHLANDS AND ISLANDS MEDICAL SERVICE BOARD.

### Increasing Failure of Medical and Nursing Services.

The fourth annual Report of this Board, dealing with the year 1917, has been presented to Parliament, and, as in previous years, we have the pleasure of presenting a summary of the Report to our readers. Like so many other schemes and activities, the work of the Board has suffered from the delaying and paralysing influences of the war. It has been impossible to carry into action the policy adopted at the outset, or at least it has been impossible to carry it into anything like full action. By the Act of Parliament (1913) which created the Board it was contemplated that the powers of the Board should cease at December 31, 1917, the expectation no doubt being that by this date the improved Medical and Nursing Services for the Highlands and Islands would be in full working order. An unkind fate having denied this expectation, it has been necessary to prolong the life of the Board, and this has been secured by the Expiring Laws Continuance Act, 1917, by the provisions of which the Board will continue in existence until December 31, 1918. Whether, in view of the national situation, any substantial advance on the present situation is possible by this date may be doubted, and the probability would seem to be that what happened in 1917 would have to be repeated in the present session of Parliament. The work of the Board has been decidedly beneficial, and has made definite advances. It cannot be allowed to halt at a half-way house, and Parliament will doubtless see that an opportunity is given for its full fruition.

Whatever may be said of the pre-war supplies of medical practitioners to the country generally, there has always been an inadequate service in the Highlands and Islands, or, at least, the service has reached a bare minimum. The scanty and scattered population, the absence of any considerable proportion of the more wealthy classes, and the limitation of ready means of communication are sufficient to explain the unattractive characters of these districts to the average medical practitioner. Upon this "bare minimum" of supply the demands of the war have fallen with truly serious effect. The fiery cross does not travel through the Highlands and Islands in vain, and the ancient tradition has maintained itself on the present occasion. The doctors, like their fellow clansmen, have felt the appeal, and who will be so bold as to challenge their decision? Still there remains the hard position of those who are left behind, for sick folk at home do not cease because men are fighting on three continents. Such a state of matters could not fail to excite the concern of a Board charged with the duty of improving medical service in these northern lands, and no vivid imagination is necessary to picture the appeals that will have been made to these authorities. Improvement in the shape of more workers was obviously, for the time being, impossible. All that could be hoped for was a reasonable approximation to the *status quo ante bellum*. Practices left vacant have been, as circumstances permit, carried on by remaining colleagues, and doctors who have left the Army after a term of service and others have been in limited measure secured. But the position is not satisfactory, and the Board finds itself almost, if not quite, at the end of its resources. A double tide of work may be possible to a practitioner for a limited period, but it cannot be long continued, and some of the Highland doctors have found this out to their cost. Even for temporary appointments the Board acknowledges it is now almost impossible to find suitable men. Here, as in so many other directions, the future is governed by the duration of the war. With the Army claiming more and more doctors, with practitioners up to fifty-six years of age undergoing medical examination relative to their fitness for service under the new Military Service Act, what chance is there that more doctors can be expected in the Highlands and Islands? Like the country generally, these districts, for the time being, are condemned to a short supply, and, of course, the deficiency is all the more conspicuous in a countryside where, even in normal circumstances, the supply is anything but an abundant one.

What is true of the medical services is true also of the nursing services. The claim of the Army thrusts home claims into the background, and it is now almost as difficult to secure qualified nurses for the Highlands as it is to obtain doctors. Equally, too, there is no prospect of improvement in this respect. Neither doctors nor nurses can be summoned into existence by stamping on the ground. From some newspaper correspondence we gather that the policy of the Highlands Board is to employ only fully-qualified nurses, and, so long as these can be obtained, the policy must command approval. But it may be suggested that in war-time some relaxation of the strict rule may be justified. There are, we believe, institutions in Scotland where women receive one year's training and acquire the certificate of the Central Midwives Board, and from these institutions a certain number of nurses go to take up work in the Highlands or elsewhere. It seems that since the Highlands Board came into operation this class of nurse has not been recognised by the Board, and for this position there is an obvious reason. But we are informed that such nurses are available, and in view of the exceptional circumstances it would seem well to consider the advisability of employing them. A nurse who has had some training and is a certificated midwife is surely better than no nurse at all, and there is the further safeguard that for the most part the nurse has to act, not on her own responsibility but under the direction of a medical practitioner.

